# Human rhinovirus infection in young African children with acute wheezing

**DOI:** 10.1186/1471-2334-11-65

**Published:** 2011-03-15

**Authors:** Heidi E Smuts, Lesley J Workman, Heather J Zar

**Affiliations:** 1 Division Medical Virology/NHLS, Department of Clinical Laboratory Sciences, University of Cape Town, Cape Town, South Africa; 2 Division of Clinical Pharmacology, Department of Medicine, University of Cape Town, Cape Town, South Africa; 3 Department of Paediatrics and Child Health, University of Cape Town, Red Cross War Memorial Children's Hospital, Cape Town, South Africa

## Abstract

**Background:**

Infections caused by human rhinoviruses (HRVs) are important triggers of wheezing in young children. Wheezy illness has increasingly been recognised as an important cause of morbidity in African children, but there is little information on the contribution of HRV to this. The aim of this study was to determine the role of HRV as a cause of acute wheezing in South African children.

**Methods:**

Two hundred and twenty children presenting consecutively at a tertiary children's hospital with a wheezing illness from May 2004 to November 2005 were prospectively enrolled. A nasal swab was taken and reverse transcription PCR used to screen the samples for HRV. The presence of human metapneumovirus, human bocavirus and human coronavirus-NL63 was assessed in all samples using PCR-based assays. A general shell vial culture using a pool of monoclonal antibodies was used to detect other common respiratory viruses on 26% of samples. Phylogenetic analysis to determine circulating HRV species was performed on a portion of HRV-positive samples. Categorical characteristics were analysed using Fisher's Exact test.

**Results:**

HRV was detected in 128 (58.2%) of children, most (72%) of whom were under 2 years of age. Presenting symptoms between the HRV-positive and negative groups were similar. Most illness was managed with ambulatory therapy, but 45 (35%) were hospitalized for treatment and 3 (2%) were admitted to intensive care. There were no in-hospital deaths. All 3 species of HRV were detected with HRV-C being the most common (52%) followed by HRV-A (37%) and HRV-B (11%). Infection with other respiratory viruses occurred in 20/128 (16%) of HRV-positive children and in 26/92 (28%) of HRV-negative samples.

**Conclusion:**

HRV may be the commonest viral infection in young South African children with acute wheezing. Infection is associated with mild or moderate clinical disease.

## Background

Wheezing is a frequent manifestation of lower respiratory tract infection (LRTI) in infants and young children. Viral infections are the commonest cause of acute wheezing. Several respiratory viruses, including respiratory syncytial virus (RSV), influenza viruses, parainfluenza viruses, enteroviruses, human coronaviruses, human metapneumovirus and human bocavirus have been associated with wheezy illness [1-5].

With the improvement of molecular techniques the frequency of HRV detection in clinical samples has increased dramatically [6-8] providing increasing evidence that HRV infection may be associated with LRTI including bronchiolitis, pneumonia, asthma exacerbations or influenza-like illnesses [9]. A recent population-based study showed that HRV was detected in 26% of children under 5 years of age hospitalized with respiratory symptoms or fever [10]. Subsequently, studies in high income countries have confirmed the importance of HRV as a cause of severe LRTI in young children requiring hospitalization [11,12]. It is unclear if the diverse spectrum of clinical illnesses associated with HRV infection is related to host factors, the infecting HRV type or both. Recent evidence suggests that infection with HRV-C may result in more severe disease [12-16].

Since the first isolation of HRV in 1953 [17] approximately 100 serotypes have been described and new types are being discovered indicating that this genus is considerably more varied than previously recognized. Based on sequence analysis, antiviral susceptibilities and receptor usage HRV was until recently divided into 2 groups; HRV-A and HRV-B [18]. However, a third and possible fourth grouping, HRV-C and HRV-D, have been identified after sequence analysis of HRV types identified some which did not cluster with HRV-A, HRV-B or other species within the genus *Enterovirus *[15,16,19-23]. HRV-C has a global distribution, with a prevalence intermediate with HRV-A and HRV-B [24].

The importance of HRV as a cause of acute wheezing illness in infants and young children has not been studied in African children. The aim of this study was to investigate the prevalence of HRV in African children with acute wheezing.

## Methods

### Study design

A prospective study of children aged 2 months to 5 years presenting with acute wheezing to Red Cross War Memorial Children's Hospital (RCCH) from May 2004 to November 2005 (2 winter seasons) was undertaken. RCCH is a public paediatric tertiary hospital in Cape Town, South Africa that provides care to children mostly from poor socio-economic backgrounds. Children were eligible if they had a history of cough or difficulty breathing within the prior 5 days and expiratory wheezing on auscultation or hyperinflation of the chest. Exclusion criteria were known underlying cardiac or chronic pulmonary disease (other than asthma), presence of stridor or daily treatment with oral corticosteroids for more than 2 days prior. Eligible children were sequentially enrolled from Monday to Friday during working hours. Clinical and sociodemographic information were recorded. Written, informed consent was obtained from a parent or guardian. The study was approved by the Human Research Ethics Committee of the Faculty of Health Sciences, University of Cape Town, South Africa.

### Nasal samples

A nasal swab was obtained using a dry sterile cotton swab inserted sequentially into each nostril to a depth of 2-3 cm and slowly withdrawn in a rotating motion as recommended by the WHO guidelines on the collection of human specimens [25]. The tip of the swab was placed in viral transport medium and transported to the Virology laboratory on the same day. After a clarification step (2000 rpm for 7 minutes) the medium was stored at -20°C.

### Rhinovirus detection

RNA was extracted from 200 μl of the respiratory sample using the Talent Seek Viral RNA kit (Talent Sri, Trieste, Italy) according to the manufacturer's instructions. The purified RNA sample was converted into cDNA using random primers (Roche Diagnostics GmbH, Mannheim, Germany) and the iScript cDNA synthesis kit (Bio-Rad, CA, USA). Samples were screened for HRV using PCR primers targeting the 5' untranslated region (5'UTR) which had previously been shown to amplify all known serotypes and HRV-C species [22]. In the first reaction 10 μl cDNA was added to a 50 μl PCR mix containing 2 IU Supertherm polymerase (JMR Holdings, Kent, UK), 15 mM Tris-HCl (pH 8), 50 mM KCl, 1.5 mM MgCl_2, _200 μmol/L each dNTP (Roche Diagnostics GmbH, Mannheim, Germany) and 0.2 μmol/L P1-1 and P3-1 primers [22]. Amplification was performed on a Thermo Hybaid PxE 0.2 thermal cycler (Thermo Scientific, Waltham, MA, USA), with the following conditions: 1 cycle of 94°C for 2 minutes, 40 cycles of 94 °C for 15 s, 50°C for 25 s, and 72°C for 35 s, and a final elongation step at 72°C for 7 minutes. The second round semi-nested PCR was performed on 2.5 μL outer PCR product using the same basic master mix ingredients containing 0.2 μmol/L P1-1, P2-1, P2-2, and P2-3 primers [22]. Cycling conditions were as for the first round PCR with an increase in annealing temperature to 55°C. Amplified products were separated by electrophoresis in 2% agarose gel, and visualized under UV irradiation after staining with ethidium bromide. The expected sizes of the outer and inner HRV PCR products were 390 bp and 300 bp respectively. VP4/VP2 amplification was performed as above on a selection of HRV-C and HRV-A samples to differentiate HRV-Ca and HRV-Cc variants using the primers described by Huang et al. [19]. All work was performed in an ISO-15189 accredited molecular laboratory which employs strict precautions to prevent contamination.

### Detection of other respiratory viruses

A general shell vial culture using a pool of monoclonal antibodies detecting respiratory syncytial virus (RSV), influenza A and B viruses, adenovirus and parainfluenza viruses 1, 2 and 3 was performed on every 4^th ^sample (n = 58) by an indirect immunofluorescence assay (Light Diagnostics, Chemicon International, CA, USA). Further specific virus identification on pool-positive samples was not undertaken. All samples were screened for human metapneumovirus (hMPV), human coronavirus-NL63 (HCoV-NL63) and human bocavirus (HBoV) by RT-PCR and PCR as previously described [26].

### Sequencing and Phylogenetic analysis

The HRV 5'UTR and VP4/VP2 PCR products were purified with a QIAquick PCR purification kit (Qiagen, Hilden, Germany) and sequenced on an ABI 310 sequencer with a fluorescent dye terminator kit (Applied Biosystems, Foster City, CA, USA). The nucleotide sequences were aligned with known HRV sequences from GenBank, including HRV Ca and Cc reference strains [27], using CLUSTALX software. A neighbour-joining phylogenetic tree was constructed using the Treecon software program (version 1.3b) with 500 bootstrap resamplings [28]. GenBank accession numbers are HM623207-HM623277.

### Statistical analysis

Continuous variables were expressed as median and inter quartile ranges and compared using Kruskal-Wallis Test. Categorical characteristics were analysed using Fisher's Exact test. A p-value of < 0.05 was considered statistically significant.

## Results

Two hundred and twenty children, median (25^th^-75^th ^percentile) age 12.2 (6.2-27.5) months, were enrolled and had a nasal swab taken. More than half, 114 (51.8%), were under 12 months, while 163 (74.1%) were less than 24 months of age, Table 1. HRV was detected in 128 (58.2%) of the samples. Most HRV-positive children were under 24 months of age (92/128; 71.8%) while half were under 12 months. There was no difference in the previous number of wheezing episodes in HRV-positive compared to negative children. The clinical symptoms and HRV status of children admitted to hospital and those treated as an out-patient is presented in Table 2. Wheezing was more common in hospitalized children (93%) and the total duration of symptoms was shorter.

**Table 1:** Clinical and demographic details of HRV-positive and negative children

	HRV-positive n = 128 (58.2%)	HRV-negative n = 92 (41.8%)	OR (95% CI)	ρ-value
Age (months) (median & IQR)	13.3(6.0-28.2)	11.7(6.7-24.6)	1.00(0.99-1.02)	0.68
Male n (%)	76 (59)	52 (41)	0.89(0.52-1.53)	0.67
Clinical symptoms n (%)				
Cough	114 (89)	83 (90)	1.13(0.47-2.74)	0.78
Wheeze	108 (84)	79 (86)	1.13(0.53-2.40)	0.76
Breathing difficulty	68 (53)	43 (47)	0.77(0.45-1.32)	0.35
Rhinorrhea	86 (67)	59 (64)	0.87(0.50-1.53)	0.64
Night waking	75 (59)	52 (57)	0.92(0.53-1.58)	0.76
Fever >37.5C	43 (34)	46 (50)	1.98(1.14-3.42)	0.02
Diarrhoea	17 (13)	10 (11)	0.80(0.35-1.83)	0.60
Vomiting	51 (40)	23 (25)	0.50(0.28-0.91)	0.02
Duration of symptoms (days) (median & IQR)	3 (2-7)	4 (2-7)	0.99(0.96-1.02)	0.58
Previous episodes of wheezing (median & IQR)	3 (2-6)	3 (2-10)	0.98 (0.94-1.02)	0.27
n = 0 (%)	29 (22.7)	21 (23)		
n = <3 (%)	40 (31.2)	22 (24)	0.98 (0.35-2.79)	0.97
n = ≥3 (%)	59 (46.1)	49 (53)	0.99 (0.94-1.03	0.57
Hospital admission n (%)				
General ward	45(35.2)	26(28.3)	1.38(0.77-2.46)	0.28
ICU	3(2.3)	4(4.4)	1.90(0.41-8.67)	0.41
Risk factors n (%)				
Premature birth (< 38 weeks gestation)	16(13)	19(21)	0.55(0.37-1.14)	0.10
Smoker in house	54(45)	4 (50)	1.37(0.80-2.35)	0.25
Relative with asthma	45(35)	2 (28)	1.38(0.34-1.11)	0.11

HRV = human rhinovirus; IQR = interquartile range; OR = odds ratio; 95% CI = 95% confidence interval; ρ-value = probability value

**Table 2:** Clinical profile of children admitted to hospital compared to those treated as an out-patient

	Hospitalized	Out-patient	OR (95% CI)	ρ-value
	**n = 71 (32.3%)**	**n = 149 (67.7%)**		

Clinical symptoms n (%)				
Cough	60(85)	137(91)	2.09(0.87-5-01)	0.10
Wheeze	66(93)	121(81)	0.33(0.12-0.89)	0.03
Breathing difficulty	35(49)	76(51)	1.07(0.61-1.88)	1.07
Rhinorrhea	43(61)	102(69)	1.41(0.78-2.54)	0.25
Night waking	38(54)	89(60)	1.29(0.73-2.27)	0.38
Fever >37.5C	30(42)	59(40)	0.89(0.51-1.59)	0.71
Diarrhoea	11(16)	16(11)	0.66(0.29-1.50)	0.32
Vomiting	24(34)	50(34)	0.99(0.54-1.80)	0.97
Duration of symptoms (days) (median & IQR)	3(1-4)	4(2-7)	0.94(0.90-0.99)	0.02

HRV positive status n (%)	45(63)	83(56)	1.38(0.77-2.46)	0.28

HRV = human rhinovirus; IQR = interquartile range; OR = odds ratio; 95% CI = 95% confidence interval; ρ-value = probability value

**Figure 1:**
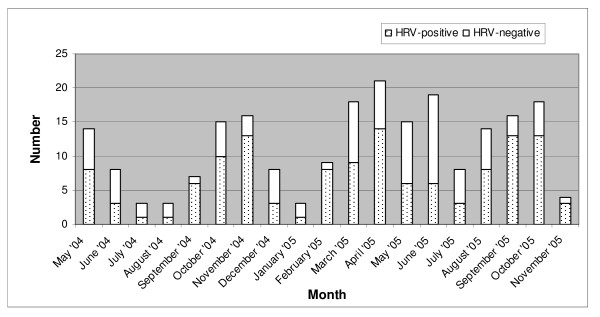
**Monthly distribution of HRV-positive and negative children**.

HRV was detected throughout the year although there was a peak in spring (September-November of 2004 and 2005) and another in February-April 2005 (autumn) (Figure 1).

Co-infection with hMPV, HBoV and HCoV-NL63 was found in 8 (6.3%), 6 (4.7%) and 1 (0.8%) of the 128 HRV-positive samples respectively. Of the 92 HRV-negative samples 5 (5.4%) were hMPV positive, 10 (10.9%) HBoV positive and 3 (3.3%) HCoV positive. Of the 58 samples screened for the common respiratory viruses by general shell vial culture 14 (24.1%) were positive; 4 (6.9%) HRV-positive and 10 (17.2%) HRV-negative.

**Figure 2:**
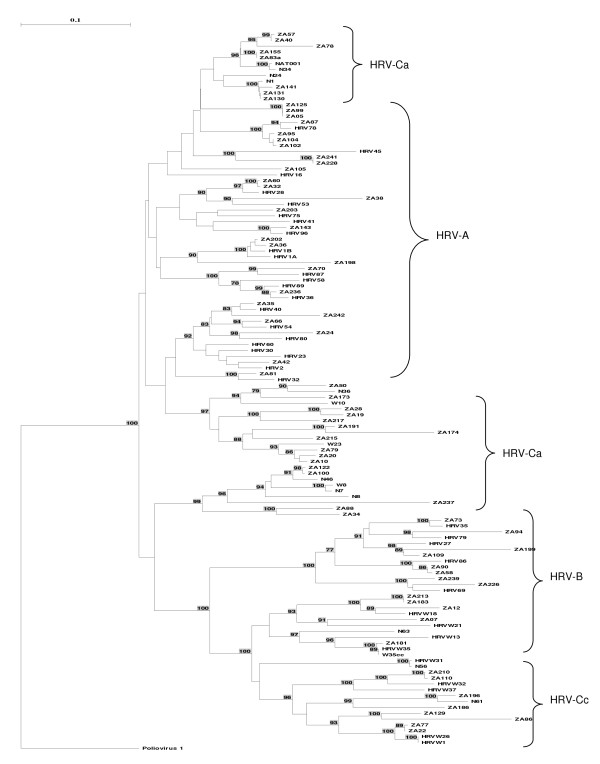
**Phylogenetic tree of the 5' UTR depicting the relationship between study samples and HRV-A, HRV-B and novel HRV-Ca and Cc species obtained from the GenBank database**.

**Figure 3:**
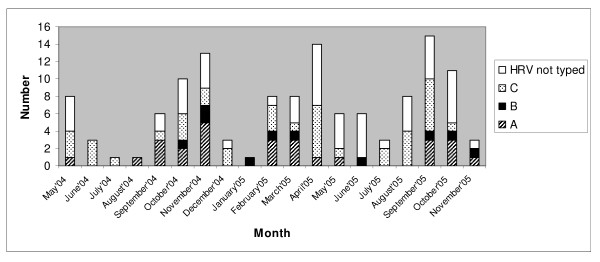
**Monthly distribution of the different HRV species**.

Sequencing of the 5' UTR was performed on 71/128 (55.5%) positive samples using convenience sampling ensuring each month of the study period was included, but without prior knowledge of patient data. Figure 2 shows the phylogenetic distribution. HRV-C can be divided into 2 variants, Ca or Cc, with either HRV-A-like or HRV-C-like 5'UTR's respectively. HRV-Ca sequences (n = 24) were interspersed within the HRV-A region of the phylogenetic tree. The remaining HRV-C samples (n = 13) formed a separate branch with the HRV-C-like 5'UTR. HRV-C was the most prevalent (37/71; 52.1%) followed by HRV-A (26/71; 36.6%) and HRV-B (8/71; 11.3%). HRV-C was found throughout the year while HRV-A had peaks in the spring and autumn seasons and HRV-B was sporadically detected (Figure 3).

Presenting symptoms or signs in HRV-infected and uninfected children were similar, except for fever which was less common in HRV-infected group of children and vomiting which was more common (Table 1). Most children with HRV infection had mild illness, although 45 (35.2%) required hospitalisation and 3 (2.3%) were admitted to the intensive care unit. The median (IQR) duration of hospitalisation was 1 (1-4) days; there was no difference in the duration of hospitalisation between HRV-infected and uninfected children. There were no in-hospital deaths. Of those admitted to hospital and where a HRV sequencing result was available (n = 15) 8 (25.8%) were typed as species C, 5 species A (16.1%) and 2 species B (6.4%).

## Discussion

HRV was the commonest viral infection in young African children presenting to a tertiary children's hospital in South Africa with acute wheezing. HRV has previously been reported in older children with wheezing or asthma exacerbations [5,29]. However, there is now accumulating evidence that HRV-associated illness occurs in young children with LRTI with many studies from other areas of the world reporting HRV detection rates from 24% to 48% [11,30,31], and as high as 78% in predisposed children [32]. Consistent with these, 72% of the HRV-positive children were under 2 years. This data is also consistent with prior studies that report mild to moderate illness in most children [11], although in this study a third required hospitalization. The study suggests that HRV is responsible for a large burden of illness in young African children with associated implications for health care utilisation.

HRV has also been associated with recurrent wheezing and with the development of asthma in older children [32-35]. HRV-induced wheezing during the first 3 years of life was associated with a 10 fold risk of developing asthma by six years in a USA cohort of infants at high risk for developing asthma [32]. Asthma is the most common chronic childhood illness in South African children affecting 10-20% of adolescents; the prevalence has increased over the last decade [36]. The role of HRV in the inception and development of asthma and in triggering acute asthma episodes in African children needs further study.

Consistent with other studies, HRV was identified throughout the year with peaks of activity in the spring of 2004 and 2005 and autumn of 2005 [3,10,35]. Interestingly this pattern was more prominent with HRV-A than HRV-C and HRV-B which were detected throughout the year or sporadically. Seasonal variation is a common feature of many respiratory viruses, including RSV, influenza, parainfluenza virus, and although the causes of this variation are largely unknown, one possible explanation may be as a result of viral interference where one respiratory virus predominates and prevents other viruses from establishing infection at the same time. As the study was of 18 months duration any inferences about seasonality are difficult to make. However, the study period included 2 winter seasons. Further long term studies of the epidemiology of viral infections and HRV specifically in African children are needed.

Phylogenetic analysis of the 5'UTR of HRV-positive samples showed the circulation of all 3 species in Cape Town, South Africa. In this study, and as reported by Huang et al. [19], HRV-C was the most prevalent followed by HRV-A and HRV-B. This distribution pattern is different from other studies where HRV-A was more frequently detected; this may vary depending on the population [21,24,37]. Consistent with previous studies HRV-C was the common type in children with wheezing and asthma [13,21,37]. Typing using the 5'UTR may underestimate the true number of HRV-C samples as there is evidence that recombination with HRV-A species at this site is a common occurrence, resulting in these types grouping with HRV-A species instead of HRV-C [19,38]. However, limited sequencing of the VP4/VP2 region (data not shown) and inclusion of sequences of HRV-C with HRV-A-like 5'UTR's in the phylogenetic analysis confirmed that HRV-C was the most common species in this population, with HRV-Ca more prevalent (65%) than HRV-Cc. Further confirmation is that all HRV-Ca sequences from this study grouped phylogenetically with GenBank-derived HRV isolates that have been confirmed to be true HRV-C types based on the classification proposed by Simmonds et al [27]. As most children presented with mild or moderate illness, and as only a subgroup of HRV isolates were sub-typed, we were unable to investigate the association between HRV species and clinical severity; this requires further investigation.

The study has some limitations. A control group of asymptomatic or clinically well children from the same time period were not included. However, previous studies have reported that HRV is more frequently found in sick children than in asymptomatic children [4,11]. HRV can be shed from the nasophaynx for 10 to 14 days in immunocompetent individuals with extended shedding in immunocompromised patients [9]. The immune status of children enrolled in this study was not recorded, although it can be inferred that some children may have been HIV-positive as South Africa is a high HIV burden country. Thus detection of HRV in some children with wheezy illness may therefore be an incidental finding due to extended shedding. Due to resource limitations, only a subset of specimens could be tested for the common respiratory viruses (RSV, influenza virus A and B, parainfluenza viruses 1, 2, 3 and adenovirus) using the relatively insensitive general shell vial culture assay which does not provide sufficient information to determine the role of co-infection and disease severity. Finally, the study did not test for all known respiratory viruses (e.g. human coronavirus 229E and OC43, and human parainfluenza type 4) and recently discovered viruses (e.g. human coronavirus HKU1 and polyomaviruses K1 and Wu) which may also play a role in wheezy illness.

## Conclusions

HRV may be the commonest virus in young African children with acute wheezing. Infection is associated with mild or moderate clinical disease. Longitudinal studies with frequent samplings and HRV type identification are needed to investigate the role of HRV in wheezing illness in African children and their long term outcome.

## Competing interests

The authors declare that they have no competing interests.

## Authors' contributions

HS conceived the study, carried out experimental studies, analyzed the sequence data and drafted manuscript. LW performed data management and statistical analyses. HZ conceived the study, obtained funding, supervised the clinical study and participated in manuscript preparation. All authors read and approved the final manuscript.

## Pre-publication history

The pre-publication history for this paper can be accessed here:

http://www.biomedcentral.com/1471-2334/11/65/prepub
